# Potential Effect of Hydroxychloroquine in Diabetes Mellitus: A Systematic Review on Preclinical and Clinical Trial Studies

**DOI:** 10.1155/2020/5214751

**Published:** 2020-02-27

**Authors:** Dawit Zewdu Wondafrash, Tsion Zewdu Desalegn, Ebrahim M. Yimer, Arega Gashaw Tsige, Betelhem Anteneh Adamu, Kaleab Alemayehu Zewdie

**Affiliations:** ^1^Department of Pharmacology and Toxicology, School of Pharmacy, Mekelle University, Mekelle, Ethiopia; ^2^Department of Pharmacy, College of Medicine and Health Sciences, Wollo University, Dessie, Ethiopia; ^3^Clinical Pharmacy Research and Course Unit, School of Pharmacy, Mekelle University, Mekelle, Ethiopia; ^4^Department of Pharmacognosy, School of Pharmacy, University of Gondar, Gondar, Ethiopia

## Abstract

**Background:**

Diabetes mellitus is a chronic metabolic disorder characterized by persistent hyperglycemia. It affects millions of people globally. In spite of many antidiabetic drugs that are available, an adequate level of control remains challenging. Hydroxychloroquine is an immunomodulatory drug that has been used for the treatment of malaria and autoimmune diseases. There is an emerging evidence that suggests its beneficial effect against diabetes mellitus. Therefore, this systematic review is aimed at discoursing the role of hydroxychloroquine against diabetes mellitus and its potential mechanisms of actions.

**Methods:**

A systematic and manual searching was carried out to retrieve relevant articles (preclinical and clinical studies) published from January 2014 to July 2019. Electronic databases including PubMed and Scopus as well as clinicaltrials.gov have been searched using different searching terms: “hydroxychloroquine,” “diabetes mellitus,” “hyperglycemia,” and “insulin resistance.” The MeSH terms (PubMed) and text words were combined with “AND” or “OR.” In addition, manual searching of Google Engine and Google Scholar was conducted. Quality assessment of all the included studies was performed using CAMARADES (preclinical studies) and the Newcastle-Ottawa Scale and Cochrane Collaboration's tools (clinical studies).

**Results:**

A total of eighteen studies (three experimental and fifteen clinical studies) were found to be eligible for the present systematic review. Among the included clinical studies (six randomized control trials, five observational studies, and four cohort studies), about 55,776 study participants were involved. Most of these studies showed significant improvement of lipid profile and insulin levels and substantial diminution of hemoglobin A1c, fasting plasma glucose, and postprandial blood glucose levels. Reduction in lysosomal degradation of the internal insulin-insulin receptor complex and enhancement in insulin sensitivity and adiponectin levels are some of the hypothesized mechanisms for the antidiabetic effect of hydroxychloroquine.

**Conclusion:**

The current review provides preliminary evidence for potential antidiabetic properties of hydroxychloroquine. Though the provided available data were promising, further clinical trials and mechanistic studies are needed to determine its long-term effects.

## 1. Introduction

Diabetes mellitus (DM) is a heterogeneous form of metabolic disorder characterized by chronic hyperglycemia with impaired carbohydrate, fat, and protein metabolism due to insulin secretion defects and/or peripheral tissue insulin action [1]. DM is one of the 21^st^ century's most extensive global health emergencies [2]. The global prevalence of DM estimated to increase, from 8.3% (366 million) in 2011 to 9.9% (522 million) by 2030. According to the report of the International Diabetes Federation (IDF), the number of people with DM in Africa will increase from 14.2 million in 2015 to 34.2 million in 2040 [3]. About half of African adults with DM live in the most populous countries: South Africa, the Democratic Republic of Congo, Nigeria, and Ethiopia [4].

The risk of diabetes-associated morbidity and mortality has been increasing unless it is treated timely [5]. Despite plenty of antidiabetic drugs in the market, adequate control of hyperglycemia remains challenging [6]. Type II diabetic patients might require insulin in addition to oral hypoglycemic agents, which is consistently augmented the side effects [7]. In addition, population-wide lifestyle modification, along with early diagnosis and cost-effective treatment of DM, is required to save lives and/or prevent devastating diabetes-related complications [2, 8].

After quinacrine was introduced as an antimalarial agent in World War II, it has associated with yellowish discoloration of the skin and other side effects. As a result of quinacrine-associated adverse effects, other antimalarial agents including chloroquine and hydroxychloroquine (HCQ) were derived with extensive chemical modification [9]. HCQ is an older drug and still used in clinical practice. It is relatively inexpensive, well-tolerated side effects, and has been used as an effective agent for management of different autoimmune disorders such as systemic lupus erythematosus (SLE), rheumatoid arthritis (RA), cancer, and various skin diseases [10–13].

Recently, there is a growing evidence that supports the beneficial effects of HCQ in DM. However, the mechanism of actions responsible for these effects still not well elucidated and may include alterations in insulin metabolism and signaling through cellular receptors [14–16]. HCQ can also minimize insulin postreceptor clearance and facilitate glucose transfer by insulin. Besides, this drug can suppress inflammatory biomarkers and control the lipid profile levels, resulting in a reduced risk of DM [17, 18]. Furthermore, reduction in hemoglobin A1c (HbA1c) fasting plasma glucose (FPG) and postprandial blood glucose (PPBG) levels are established in various studies [17, 19–22]. Therefore, this review is aimed at assessing the efficacy of HCQ therapy in experimental models of DM and diabetic patients.

## 2. Methodology

### 2.1. Data Sources and Searching Strategy

A web-based systematic search strategy for literature was employed through various electronic databases including PubMed, Scopus, and clinicaltrials.gov. The search was limited to original studies conducted on both human and animal subjects, reported in English language and published in peer-reviewed journals from 2014 to 2019. The search was conducted using MeSH terms (PubMed) and text words for each domain (diabetes mellitus and hydroxychloroquine). The search terms used were “diabetes mellitus,” “hyperglycemia,” “insulin resistance,” and “hydroxychloroquine.” The MeSH terms and/or text words were combined by “AND” or “OR” in each domain. Manual searching using Google Engine and Google Scholar was also conducted to identify other relevant studies using “hydroxychloroquine and therapeutic role in diabetes mellitus” OR “hydroxychloroquine AND diabetes mellitus” OR “hydroxychloroquine AND hyperglycemia” (Table 1). Clinicaltrials.gov was also used to check the online status of clinical studies.

### 2.2. Criteria for Selection of Studies

Preclinical and clinical studies intended to assess the possible antihyperglycemic effects of HCQ in experimentally induced DM models and human trials were included. Original articles published from January 2014 to July 2019 were included in the present review. Original articles including randomized controlled trials (RCTs) and observational and cohort studies aimed at evaluating the therapeutic role of HCQ in diabetic individuals as well as experimental models of diabetes correlated to the intervention of HCQ attempt to control chemical-induced hyperglycemia in animal diabetic models were included. In addition, the searches were restricted to articles published in English language only regardless of the sample size used and/or duration of follow-up. However, cross-sectional studies and case reports and/or partially accessed (abstract only) articles (both animal diabetic model and clinical studies) were excluded.

### 2.3. Screening and Eligibility of Studies

The studies identified from various electronic database sources were imported to Endnote software in order to sort out duplicated articles. Each of the articles retrieved was assessed by two authors (DZW and TZD) independently for eligibility of the studies by reading the title and abstract using the developed exclusion and inclusion criteria. This process was followed by the assessment and retrieval of the full texts of the relevant citations. The other authors (KAZ and EMY) were involved in resolving the disagreement between the two authors who assessed the eligibility of the articles.

### 2.4. Data Extraction Process

The extraction of data from each study was carried out using a predefined form. The two authors (DZW and TZD) independently extracted the data related to study characteristics: year of publication, study design (RCTs, cohort and observational studies), study population, sample size, follow-up duration, drug type, and outcomes of treatment documented during the follow-up period.

### 2.5. Quality Assessment of the Studies

The preclinical studies were assessed independently by the two authors (DZW and TZD) for methodological quality of the included articles in the review using the Collaborative Approach to Meta-Analysis and Review of Animal Data from Experimental Studies (CAMARADES) with slight modification of the ten-item quality assessment checklists [23, 24]. Each item was given either one point if it satisfied the criteria or zero if insufficiently described or not explained at all. In order to better understand the risk of bias among included clinical studies, the Newcastle-Ottawa Scale (NOS) was applied for quality assessment in observational and cohort studies [25]. The Cochrane Collaboration's tool was also used to assess the risk of bias in RCTs [26]. Each study was assessed for risk of bias in selection, comparability of study groups, and ascertainment of the outcome of interest.

## 3. Results

**Figure 1:**
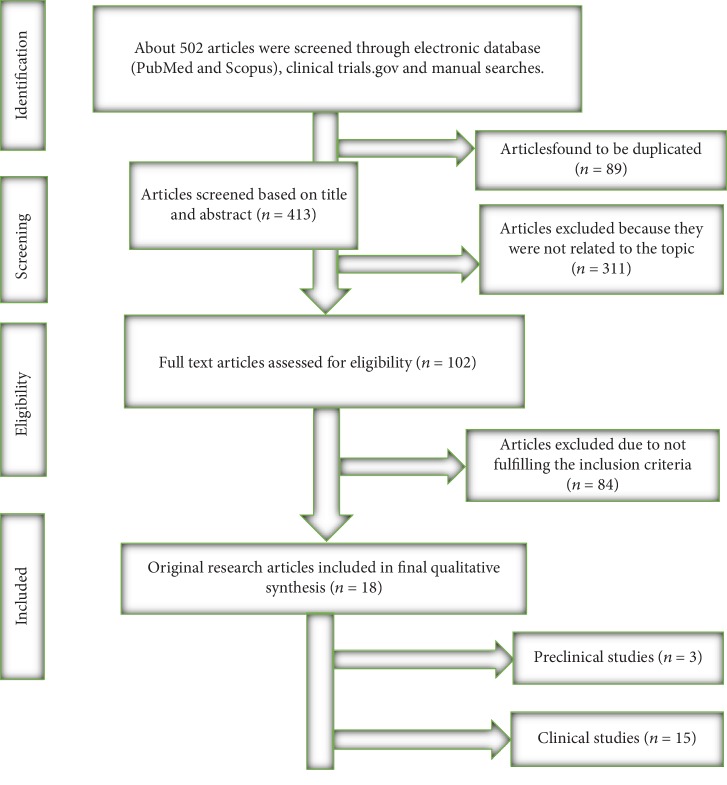
Flow diagram of the article screening process for qualitative analysis.

There were 502 potentially eligible articles that were identified by systematic and manual searching strategy, of which, 89 were excluded due to duplication. After a screening of the articles by title and abstract, another 413 articles were excluded. Finally, a total of eighteen original articles were included for qualitative analysis (Figure 1). Among the included studies, three were animal studies while the rest were human trials that involved a total of 55,776 participants.

The quality assessment of each experimental study was performed according to CAMARADES, and the average quality scores for all the included articles were greater than or equal to five ([Supplementary-material supplementary-material-1]). All the included cohort and observational studies scored greater than or equal to five based on the NOS score ([Supplementary-material supplementary-material-1]). Besides, the assessment of the risk of bias of the RCT studies was performed using the Cochrane Collaboration's tools ([Supplementary-material supplementary-material-1]). From the fifteen clinical studies, six were RCTs, five were observational studies, and the rest four articles were retrospective cohort studies. About eleven studies are aimed at evaluating the role of HCQ in type II DM patients while a single study was conducted on newly diagnosed SLE patients to assess the effect of HCQ in preventing the development of DM, and another study was carried out on a prediabetic condition.

## 4. Therapeutic Role of Hydroxychloroquine in Diabetes Mellitus

### 4.1. The Effects of Hydroxychloroquine in Experimental Diabetes Mellitus

Several experimental studies have been conducted regarding the efficacy and safety profile of HCQ in different animal models. However, only three original articles conducted in experimentally induced diabetic models that fulfill the selection criteria were selected. Accordingly, the potential effect of HCQ in glycemic control has been presented in Table 2.

### 4.2. The Effects of Hydroxychloroquine on Patients with Diabetes Mellitus

HCQ therapy showed an improvement of glycemic level in diabetic patients that could be a new therapeutic approach for the treatment of DM. According to a study done by [30], a combination of HCQ and insulin decreased the HbA1c level in patients with type II DM as compared to insulin therapy with other oral hypoglycemic agents. In addition, HCQ to insulin therapy is also associated with a reduced incidence of hypoglycemia. A number of clinical studies demonstrated the safety and efficacy of HCQ and were presented in Table 3.

## 5. Discussion

In alloxan-induced diabetic rats, an experimental study was done to investigate the antihyperglycemic effect of HCQ and atorvastatin. The study reported that a high dose combination of HCQ (10 mg/kg) and atorvastatin (200 mg/kg) exhibited the highest reduction (21%) in blood glucose levels than low dose combinations and individual treatments [39]. Another experimental study suggested that compared to monotherapy, a combination of HCQ with oral antihyperglycemic drugs (metformin and glibenclamide) significantly decreased blood glucose level and improved lipid profiles [27]. Improved insulin sensitivity and insulin response were observed 30 min after insulin injection combining TAD with HCQ [29]. According to a study done by [28], HCQ inhibits inflammatory cytokines and protects against *β* cell loss.

Hyperglycemia, which was uncontrolled with a combination of the optimum dose of metformin and glimepiride, experienced better glycemic benefit treated with 400 mg of HCQ for 24 weeks [20]. According to Baidya et al., HCQ-treated group showed a significant reduction of HbA1c level and insulin requirement in a dose-dependent manner [36]. In another study, the coadministration of HCQ 400 mg with insulin results in a sustained improvement in glycemic control when administered to patients with type II DM who were poorly controlled on insulin therapy along with other oral antidiabetic agents. Furthermore, these findings are similar to the results of other studies suggesting that HCQ improves glycemic control by increasing insulin sensitivity in patients with type II DM [20, 22, 29, 30, 36].

Two studies have compared the safety and efficacy of teneligliptin with HCQ. According to Singh *et al*. [22], substituting teneligliptin with HCQ reduced HbA1c level by (-1.1%) from the baseline and total cholesterol and LDL level was decreased while significantly increased the level of HDL. Besides, good cholesterol and glycemic control were noticed in prolonged use of HCQ and showed kidney protecting effect.

Prediabetes is considered as an intermediate clinical condition without complete definitive criteria of diabetes, but with blood glucose higher than the normal range. Patients with a prediabetes state are at increased risk for clinical DM and are also predisposed to various complications including cardiovascular disorders. Treatment with HCQ can be employed as a therapeutic option in patients with prediabetic states who are risky of developing DM [18].

In an RCT-based study, 39 prediabetic patients who received 6.5 mg/kg/day HCQ for 12 weeks showed a significant increment of insulin level and reduction of blood glucose level, and the hypoglycemic effect of HCQ also did not affect body organs and metabolic components [18]. A cohort study conducted by Chen *et al*. [33] found that the risk of DM was low for RA or PS/PSA patients initiated with an anti-TNF agent and concomitant HCQ therapy followed by those who used HCQ without an anti-TNF agent. Another cohort study also reported that type II DM patients with SLE showed a significantly lower probability of developing overt DM at a cumulative dose of ≥129 g HCQ use [31].

A total of 6 articles were identified through on the clinicaltrials.gov, and only three of them were found to be duplicated, and the result of two studies has been clearly described in Table 3. Whereas, for the rest of the studies, the outcomes of their trials have not posted yet.

In one study, type I DM patients undergoing total pancreatectomy and autologous islet transplantation (TPAIT) for chronic pancreatitis consist of 5 patients treated with HCQ (200 mg/day) 30 days prior and 3-month postsurgery and 5 placebo-treated patients following the same schedule as HCQ taking group is still under investigation (phase II trial) to investigate the islet cell function and metabolic performance of the HCQ Vs placebo-treated groups (https://clinicaltrials.gov/ct2/show/NCT03283566).

In the other study, 201 type I DM patients were randomly allocated into HCQ and placebo groups (2 : 1) to study if HCQ can help prevent or delay the progression from normal glucose tolerance (stage 1) to abnormal glucose tolerance (stage 2) or type I diabetes (stage 3), this is also on phase II trial estimated to be completed in August 2024 (https://clinicaltrials.gov/ct2/show/NCT03428945).

In addition, type II DM involving 30 participants who were on a maximum dose of metformin was randomly allocated to HCQ (200 mg/day) and placebo taking groups in order to investigate the metabolic effect of HCQ. The estimated primary completion date of this study is in December 2020 (https://clinicaltrials.gov/ct2/show/NCT02026232).

Daily dose > 400 mg/day, or >6.5 mg/kg ideal/lean body weight for short individuals; cumulative dose > 1,000 g; duration of use > 5 years; renal or hepatic dysfunction; obesity; age > 60 years; and preexisting retinal disease or maculopathy are risk factors for toxicity of HCQ [40, 41]. HCQ is the safest DMARD, but gastrointestinal discomfort and pruritus have been among the commonly reported side effects [42]. Because of the promising antidiabetic efficacy, relative safety, and low cost of HCQ, it can emerge as a valuable therapeutic option in the management of type II DM patients uncontrolled by conventional oral therapies [21].

## 6. Possible Molecular Mechanisms of Hydroxychloroquine in Diabetes Mellitus

HCQ has multiple therapeutic properties, many of them not being fully understood at a molecular level. But its therapeutic effect has been found to possess anti-inflammatory, immunomodulating, anti-infective, and antithrombotic actions [43].

HCQ inhibits the degradation of insulin enhancing the metabolic effects of the hormone to improve insulin sensitivity [44]. The metabolic effect of HCQ is reducing dissociation of insulin from its receptor (tyrosine kinase) and increase the biologic half-life of insulin receptor complex which prolongs the action of insulin [16].

The possible explanation for the glucose-lowering effect of HCQ maybe that HCQ stabilizes intracellular lysosomes and slows the breakdown of the internalized insulin receptor complex [21] HCQ is an acidotrophic agent when intracellular concentration of HCQ reaches high, intracellular pH is raised causing inactivation of proteolytic enzyme (insulinase) that is responsible for degradation of insulin-resulting recirculation of substantial proportion of insulin in the active form [14].

It has been shown that during inflammation, cytokines such as tumor necrosis factor *α* (TNF*α*) and interleukin-6 (IL-6) increased adiposity and insulin resistance by triggering key steps in the insulin signaling, hence influencing insulin and glucose metabolism [45]. TNF*α* decreases tyrosine phosphorylation of the insulin receptor and insulin receptor substrate-1 (IRS-1) kinase and induces serine phosphorylation of IRS-1, which becomes an insulin receptor inhibitor in adipocytes and skeletal muscle cells. Thus, instead of acting only as a substrate for the insulin receptor, IRS-1 induces a negative feedback loop that declines the enzymatic activity of the receptor, thereby inhibiting its signaling pathway [46].

A retrospective cohort study done on patients' diagnosis with either of RA or psoriasis treated with TNF*α* inhibitors, methotrexate, HCQ, and other nonbiologic DMARDs reported the reduced relative risk of DM for TNF*α* inhibitor and HCQ compared with other nonbiologic DMARDs [44]. Another retrospective cohort study conducted by Ozen and his coworkers on a total of 13,669 RA patients with and without incident DM were grouped into (i) methotrexate monotherapy, (ii) abatacept with or without synthetic DMARDs, (iii) any other DMARDs with methotrexate, and (iv) all other DMARDs without methotrexate, along with separate statin, glucocorticoid, and HCQ. RA patients who were on HCQ treatment showed a significant risk reduction of developing DM compared to groups who were not on HCQ treatment [47].

HCQ increases insulin sensitivity and reduces insulin resistance through its indirect effect by reducing inflammation [48]. HCQ has been reported to improve insulin sensitivity through the activation of protein kinase *β* (Akt) resulting in increased glucose uptake and glycogen synthesis [49].

## 7. Conclusion

In general, HCQ can lead to significant and clinically meaningful improvements in glycemic control with diabetic patients. Although different mechanisms for HCQ in type II DM have been proposed, available shreds of evidence are preliminary; further mechanistic, efficacy, and safety-related preclinical and clinical studies are still necessary to verify the usefulness of this agent in treating DM.

## Figures and Tables

**Table 1:** Techniques of extraction and key terms used during data extraction of articles.

Databases and search terms used to extract all available and relevant articles	Number of articles retrieved (*n*)
PubMed	
(1) “Diabetes mellitus [Mesh] OR diabetes mellitus^∗^[Tw]”	
(2) “Hyperglycemia [Mesh] OR hyperglycemia^∗^[Tw]”	
(3) “Insulin resistance [Mesh] OR insulin resistance^∗^[Tw]”	
(4) “Hydroxychloroquine [Mesh] OR hydroxychloroquine^∗^[Tw]”	
Then, search for (#1 OR #2 OR #3 AND #4)	*n* = 98
Scopus	
“Diabetes mellitus” OR “hyperglycemia” AND “hydroxychloroquine”	*n* = 159
Clinicaltrials.gov: condition “diabetes mellitus,” other terms “hydroxychloroquine”	*n* = 6
Gray kinds of literature searching (Google Scholar and regular Google Engine) (1) Antidiabetic activity of hydroxychloroquine OR diabetes mellitus and hydroxychloroquine (2) Antihyperglycemic effect of hydroxychloroquine OR hyperglycemia and hydroxychloroquine	239

**Table 2:** Effect of hydroxychloroquine against diabetes mellitus on different animal models.

Experimental model	Method and intervention	Treatment outcome	References
Alloxan-induced diabetic rats	Alloxan- (120 mg/kg) induced 42 diabetic rats were randomly allocated to seven groups taking metformin 850 mg/70 kg body weight (BW)/day, glibenclamide at 10 mg/70 kg BW/day, HCQ 300 mg/70 kg BW/day, individually for 2 weeks and metformin 850 mg/70 kg BW/day, glibenclamide 10 mg/70 kg BW/day, HCQ 300 mg/70 kg BW/day, combination therapy, metformin with HCQ (425 mg/70 kg BW + 150 mg/70 kg BW) and glibenclamide with HCQ (5 mg/70 kg BW + 150 mg/70 kg BW), respectively, for 4 weeks.	Combination of HCQ with metformin and glibenclamide significantly reduced blood glucose level than individual therapy. In addition, increment in liver glycogen levels was observed. Further improvement in the lipid profile was observed in combination therapy.	[27]
Chow and HFD-received *Sprague Dawley* rats	54 rats were randomly divided into three groups (*n* = 18): group I: control group; group II: high-fat diet (HFD) group; and group III: HCQ+HFD group. Taking 6.5 mg/kg/d orally of HCQ 200 mg tablet for 12 weeks.	Mild degenerative change in the islet of Langerhans (IOL) was seen in the HFD group, and it was preserved in the HCQ taking group. Serum levels of FBG, insulin, AUC, HOMA-IR significantly decreased in the HFD+HCQ group compared with those of the HFD group. Adipokines were significantly elevated in the HFD groups.	[28]
Adult male mice	76 mice were randomly assigned into four groups (*n* = 19/group): group I, control vehicle (0.5% hydroxypropyl methylcellulose and 1% Tween 80 in sterile water); group II, TAD, 6 mg/kg daily mixed in-vehicle solvent; group III, HCQ 50 mg/kg daily mixed in-vehicle solvent; and group IV, Tadalafil (TAD) (6 mg/kg daily) 1 HCQ (50 mg/kg daily) mixed in-vehicle solvent.	The plasma level of insulin was increased, and the plasma level of glucose was decreased in the HCQ and TAD±HCQ groups. Plasma levels of free fatty acids and triglycerides were significantly decreased in the TAD±HCQ group. TAD±HCQ improved insulin TAD, HCQ, and TAD±HCQ treated animals had a clear trend toward increased pancreas mass/body weight. The mammalian target of rapamycin complex 2 was dramatically increased in the HCQ and TAD±HCQ treatment groups.	[29]

AUC: area under the curve; BW: body weight; HCQ: hydroxychloroquine; HFD: high-fat diet; HOMA-IR: homeostatic model assessment-insulin resistance; IOL: the islet of Langerhans; TAD: Tadalafil.

**Table 3:** Therapeutic effect of hydroxychloroquine in human study.

Study design	Method and intervention	Main findings	References
Population-based cohort study	Excluding patients with prior history of RA, DM, and PSA, 8628 newly identified SLE patients who are taking HCQ and glucocorticoids were deemed to be eligible for the study.	HCQ reduces the risk of DM in a dose-dependent manner. Compared to groups that took HCQ dose less than 129 g, those who took HCQ greater than 129 g had a significantly lower probability of developing DM.	[31]
Phase II double-blind, randomized control trial	Eligible 15 nondiabetic rheumatic arteries patients were allocated to placebo and 13 to HCQ groups (400 mg) for 13 weeks.	ISI is increased in HCQ but not in the placebo group. The beta-cell function also improved in the HCQ group, not in placebo. HCQ showed modest improvement in fasting plasma glucose concentration and HbA1c level. No significant change has been observed in circulatory biomarkers, but adiponectin was increased in the HCQ group.	[32]
Randomized double-blind control trial	A sum of 20 prediabetic patients who meet the inclusion criteria was allocated to the HCQ group and 19 in the placebo group intervened for 12 weeks.	Level of insulin increased from 12.3 ± 10.6 to 78.3 ± 53.5 units in the case group and from 9.8 ± 5.3 to 40.8 ± 31.4 units in the control group, HCQ. Those taking HCQ experience reduction of glucose at 60 minutes of OGTT.	[18]
Open-labeled comparative observational study	A total of 100 type II DM patients who were uncontrolled with a combination of antidiabetes medication were grouped into group one receiving metformin, glimepiride, and teneligliptin, and the other group were receiving metformin, glimepiride, and HCQ for 24 weeks.	At week 24, the HbA1c level is decreased by -1.6% in group one and by -1.8% in group two from the baseline. Moreover, FBG and PPBG showed a significant reduction in group two who were on HCQ treatment.	[20]
Retrospective cohort study	36,329 AS, RA, or PS/PSA patients taking those drugs were enrolled into four mutually exclusive groups. (i) anti-TNF*α* with or without DMARD, (ii) CSA without anti-TNF*α* or HCQ, (iii) HCQ without anti-TNF or cyclosporine, and (iv) other nonbiologic DMARD without anti-TNF, CSA, or HCQ.	Newly diagnosed DM was not observed among those given anti-TNF+HCQ therapy. In the RA group, anti-TNF+HCQ therapy and HCQ alone had significant protective effects. In the PS/PSA group, HCQ had a significant protective effect.	[33]
Randomized double-blind study	267 uncontrolled type II DM patients (HbA1c ≥ 7.5% and ≤11.5%), post 3 months of treatment with glimepiride/gliclazide and metformin, to additionally receive HCQ 400 mg/day (*n* = 135) or pioglitazone 15 mg/day (*n* = 132) for 24 weeks. Efficacy of HCQ was compared with pioglitazone by changes in HbA1c, FBG, and PPG blood glucose at week 12 and week 24.	At week 12 and week 24, HbA1c, FBG, and PPG significantly reduced from baseline in both groups. The mean reduction in glycemic parameters at week 12 was not significantly different between the HCQ and pioglitazone groups. Change in total cholesterol (TC) and LDL-C was significant in favor of HCQ. Triglycerides significantly reduced in both groups at week 24. Mean HDL-C remained unchanged.	[17]
Multicenter open-labeled comparative observational study	A total of 240 type II DM patients who were on combination treatment of insulin, glimepiride (1 to 4 mg), and metformin (500 to 2,000 mg) with poor glycemic control were randomly allocated to group I, HCQ 200 mg (*n* = 120), or group II, HCQ 400 mg (120) once daily for 24 weeks.	HbA1c level is significantly reduced by 0.8% and 1.3% in groups I and II, respectively, from the baseline, and the total mean daily dose of insulin was significantly reduced. The effect of HCQ in type II DM patients is dose-dependent; the higher the dose of HCQ, the greater reduction in HbA1c level will be achieved. The reduction of hs-CRP by greater than or equal to 1 is correlating with the reduction of HbA1c in a range of 0.8% to 1.3%.	[19]
A multicenter retrospective cohort study	Poorly controlled type II DM patients (*n* = 500) treated with teneligliptin-based regimen (20 mg) per day were replaced by 400 mg HCQ along with metformin and glimepiride, and their data were deemed to be eligible for analysis the whole 24 weeks.	The addition of HCQ in place of teneligliptin has led to a significant reduction of HbA1c from baseline to 24 weeks. FBG and PPBG level was also decreased. It has been seen that total cholesterol, triglycerides, and LDL levels were decreased and HDL levels were significantly increased. Apart from providing tight glycemic control, this significant reduction in lipid profile also indicates that HCQ has added an advantage in reducing cardiovascular risk.	[22]
Multicenter, open-label, parallel-group observational study	Patients who were on a maximum dose of metformin and glibenclamide inadequately controlled with insulin therapy were deemed to be eligible: 338 type II DM patients were randomly allocated to the HCQ group taking 400 mg QD and 343 to the sitagliptin group taking 100 QD for 24 weeks.	Compared to sitagliptin, combination of insulin and HCQ significantly improves FPG and PPG. HBA1c less than seven was achieved when HCQ was added to insulin therapy. In the HCQ group, a daily dose of insulin was significantly reduced. The rapid deterioration of glycemic control was observed so close monitoring or aggressive therapy for normoglycemic effect is mandatory.	[21]
Real-world observational study	A total of 640 eligible type II DM patients were randomly grouped to HCQ 400 mg/day, metformin 1,000 mg/day and glimepiride 2 mg/day taking groups and to sitagliptin group taking 100 mg/day, 1,000 mg/day metformin, and 2 mg/day glimepiride for 24 weeks.	Significant improvement in PPBG, FBG, and HbA1c level was found. After 24 weeks, 1.4% HbA1c level reduction was observed in the HCQ group, 1.2% with sitagliptin. At week 24, both QUICKI and HOMA-IR were significantly changed, but the favorable effect was observed in the HCQ group. Plasma hs-CRP declines more in the HCQ group than the sitagliptin group. HDL-C was markedly improved, and reduction to T-C and LDL-C was more favorable to the HCQ group. No episode of hypoglycemia exhibited marked severity.	[34]
Randomized clinical trial	A sum of 165 type II DM patients who meet inclusion criteria was randomized into group I taking metformin 2,000 mg/day with glimepiride 2 mg/day, group II taking 1,000 mg/day metformin with 4 mg glimepiride, and group III taking 1,000 mg/day metformin, 2 mg/day glimepiride with 400 mg/day of HCQ.	HbA1c level was reduced by 1.1, 1.3, and 1.5% from baseline in those taking the maximum doses of metformin, glimepiride, and HCQ, respectively. The risk of hypoglycemia was significantly lower in the first and third groups than the second group. Except for the GI side effect in the first group, no significant adverse effect has been reported in three of the groups.	[35]
Randomized, prospective, parallel-group study	Eligible 300 type II DM patients were randomly placed to the HCQ group (*n* = 148) and to teneligliptin (*n* = 152) for 24 weeks.	HbA1c level was reduced by 1.2% and 0.9% from the baseline in HCQ and teneligliptin taking group consecutively. A daily dose of insulin was significantly reduced in the HCQ taking group. Significant reduction in FPG and PPG was pronounced in HCQ taking groups.	[30]
A randomized active-controlled study	A total of 100 eligible patients were randomly allocated to the HCQ group taking 400 mg per day and the other 50 to vildagliptin taking 100 mg per day apart from metformin 1gm/day and glimepiride 2 mg/day for 24 weeks.	At week 24, HbA1c level decreased by 1.3 and 1.1% from baseline on HCQ and vildagliptin taking groups, respectively. There was a significant reduction in FPG and PPPG. Two patients in the HCQ group report for mild hypoglycemia and five patients in vildagliptin arm, but severe hypoglycemia effect has not been reported. There was no incidence of renal and hepatic toxicity.	[36]
Multicenter, open-label, observational study	Poorly controlled type II DM patients (*n* = 100) with a combination treatment of gliclazide (80 mg/day) and metformin (1,000 mg/day) along with twice a day basal insulin glargine therapy (≥30 units a day) were given to take a fixed dose of 400 mg HCQ QD for 6 months.	Beginning at one month and reaching maximal effects at six months, a significant decrease in HbA1c, FBG, and PPBG level was observed. There was no clinically significant change in ECG. Transient elevation of AST and ALT levels was not observed. Hypoglycemic symptoms that require hospitalization were not reported.	[37]
Retrospective cohort study	From a total of 7,774 newly diagnosed Sjögren syndrome (SS), 510 patients who had used HCQ for the first time for at least 90 days and who had been diagnosed with SS for no longer than 180 days were observed.	Patients treated with HCQ had a significantly lower cumulative incidence than those not treated with HCQ. Using HCQ had a significant preventive effect on the development of DM with Sjögren syndrome. The usage of HCQ was associated with a reduced risk of developing DM.	[38]

AS: ankylosing spondylitis; AST: aspartate aminotransferase; ALT: alanine transaminase; BMI: body mass index; BW: body weight; CRP: c-reactive protein; DM: diabetes mellitus; DMARD: disease-modifying antirheumatic drugs; ECG: electrocardiogram; eGFR: estimated glomerular filtration rate; FBS: fasting blood sugar; FPG: fasting plasma glucose; GI: gastrointestinal; HbA1c: hemoglobin A1c; HCQ: hydroxychloroquine; HDL: high-density lipoprotein; HFD: high-fat diet; HOMA-IR: homeostatic model assessment-insulin resistance; IOL: islet of Langerhans, ISI: insulin sensitivity index; LDL: low-density lipoprotein; mTORC2: mammalian target of rapamycin complex 2; OD: once a day; OGTT: oral glucose tolerance test; PPBG: postprandial blood glucose; PPG: postprandial glucose; PS: psoriasis; PSA: psoriatic arthritis; QUICKI: quantitative insulin sensitivity index; RA: rheumatic arteritis; RCT: random control trial; RR: relative risk; SLE: systemic lupus erythematosus; SS: Sjögren syndrome; TC: total cholesterol.

## Abbreviations

DM: Diabetes mellitus
DMARD: Disease-modifying antirheumatic drugs
FBG: Fasting blood glucose
HbA1c: Hemoglobin A1c
HCQ: Hydroxychloroquine
HDL: High-density lipoprotein
IDF: International Diabetes Federation
IRS-1: Insulin receptor substrate-1
NOS: Newcastle-Ottawa Scale
PPBG: Postprandial blood glucose
PPG: Postprandial glucose
RA: Rheumatic arteritis
SLE: Systemic lupus erythematosus
TNF*α*: Tumor necrosis factor *α.*

## Supplementary Materials

Supplementary Materials Table S1: we have conducted the quality assessment of included preclinical studies using Collaborative Approach to Meta-Analysis and Review of Animal Data from Experimental Studies (CAMARADES) according to Macleod et al. [23] and Yimer et al. [24]. The included preclinical studies were evaluated based on the ten-item quality checklists, and the maximum score for each study was ten. Finally, the overall quality score was calculated and expressed as mean ± standard error of the mean. Table S2: we have evaluated the quality of included observational and cohort studies with the Newcastle-Ottawa Scale (NOS). This scale had been adapted from Wells et al. [25]. According to the NOS scale, we evaluate selection, comparability, the outcome of studies, and the average score for each study. Table S3: we have assessed the risk of bias for the randomized control trial (RCT) studies using the Cochrane Collaboration's tool based on Higgins et al. [26]. The assessment of the risk of bias of each included RCTs study was rated as low risk of bias, unclear risk of bias, or high risk of bias.
