# Supplementary material for: Potential Effect of Hydroxychloroquine in Diabetes Mellitus: A Systematic Review on Preclinical and Clinical Trial Studies
Source: J Diabetes Res. 2020 Feb 27;2020:5214751. doi: 10.1155/2020/5214751 (PMC7064866; doi:10.1155/2020/5214751)
Supplement: Supplementary Materials — Table S1: we have conducted the quality assessment of included preclinical studies using Collaborative Approach to Meta-Analysis and Review of Animal Data from Experimental Studies (CAMARADES) according to Macleod et al. [23] and Yimer et al. [24]. The included preclinical studies were evaluated based on the ten-item quality checklists, and the maximum score for each study was ten. Finally, the overall quality score was calculated and expressed as mean ± standard error of the mean. Table S2: we have evaluated the quality of included observational and cohort studies with the Newcastle-Ottawa Scale (NOS). This scale had been adapted from Wells et al. [25]. According to the NOS scale, we evaluate selection, comparability, the outcome of studies, and the average score for each study. Table S3: we have assessed the risk of bias for the randomized control trial (RCT) studies using the Cochrane Collaboration's tool based on Higgins et al. [26]. The assessment of the risk of bias of each included RCTs study was rated as low risk of bias, unclear risk of bias, or high risk of bias. [file 5214751.f1.pdf]

Table S1: The quality assessment of included preclinical studies according to a modified CAMARADES checklist items (Macleod *et al*., 2004; Yimer *et al*., 2019).

**References**	**Criteria**										
	I	II	III	IV	V	VI	VII	VIII	IX	X	**Total** (of 10)	**Mean Quality scores**
(Zannah *et al*., 2014)	1	1	1	1	1	0	1	1	1	1	9	8.66 ± 0.33
(Abdel-Hamid and Firgany., 2016)	1	1	1	1	1	0	1	1	1	1	9	
(Wang and Kukreja., 2017)	1	1	1	1	1	0	1	1	0	1	8	

I: Publication in peer-reviewed journal, II: Number of experimental and control groups, III: Housing and Husbandry Conditions, IV: Details of intervention/ exposure group procedures, V: Random allocation to groups, VI: Concealment of allocation, VII: Blinded assessment of outcomes, VIII: Biochemical evaluations, IX: Histopathological evaluations, X: Statistical analysis 1: Criterion is satisfied, 0: Insufficiently described or not explained at all; the mean quality score is expressed as mean ± standard error of the mean (SEM) and (minimum and maximum score)

Table S2: Evaluation of quality of included observational and cohort studies by Newcastle-Ottawa scale (NOS) (Wells *et al.*, 2009)

Cohort studies (n = 4)	Selection (4)				Comparability (2)	Exposure			Total (0-9)
	Representative ness of the exposed cohort	Selection of the non-exposed cohort	Ascertain ment of exposure	Outcome of interest not present at start of study	control for important factor or additional factor	Ascertainm ent of outcome	Follow-up long enough form outcome s to occur	Adequacy of follow-up of cohorts	
Chen *et al.*, 2019	*	*	*	*	**	*	-	-	7
Singh *et al.*, 2018	*	*	*	*	**	*	*	*	9
Chen *et al.*, 2017	*	*	*	*	*	*	*	*	8
Chen *et al.*, 2015	*	--	*	*	*	*	*	*	7
Observational studies (n =5)	Selection (4)				Comparability (2)	Exposure			Total (0-9)
	Adequate definition of case	Representative ness of cases	Selection of controls	Definition of controls	Control for important factor or additional factor	Ascertainm ent of exposure (blinding)	Some method of ascertainme nt for subjects	Non-response rate	
Chandra *et al.*, 2018	*	*	*	*	*	-	*	*	7
Jagnani *et al.*, 2017	*	-	-	*	**	-	*	-	5
Baidya *et al.*, 2018a	*	*	*	*	*	*	*	-	7
Ahmed *et al.*, 2018	*	*	*	*	**	*	*	*	8
Kumar *et al.*, 2018	*	-	*	*	**	-	*	-	6

Table S3: Risk of bias for the randomized control trial studies with the Cochrane Collaboration's tool (Higgins *et al.*, 2011)

References	Random sequence generation (selection bias)	Allocation concealment (selection bias)	Blinding of participants and researchers (performance bias)	Blinding of outcome assessment (detection bias)	Incomplete outcome data (attrition bias)	Selective reporting (reporting bias)	Other bias
Sheikhbahaie *et al.*, 2016	+	+	+	+	+	+	?
Baidya *et al.*, 2018b	+	?	–	–	+	–	–
Ranjan *et al.*, 2018	+	?	–	–	?	?	?
Baidya and Ahmed, 2018	+	–	+	+	+	+	?
Pareek *et al.*, 2014	+	?	–	–	?	+	?
Wasko *et al.*,2015	+	+	+	+	–	+	–

+ Low risk of bias ? Unclear risk of bias – High risk of bias
